# Real‐time surgical tool detection with multi‐scale positional encoding and contrastive learning

**DOI:** 10.1049/htl2.12060

**Published:** 2023-12-07

**Authors:** Gerardo Loza, Pietro Valdastri, Sharib Ali

**Affiliations:** ^1^ School of Computing, Faculty of Engineering and Physical Sciences University of Leeds West Yorkshire UK; ^2^ School of Electronic and Electrical Engineering, Faculty of Engineering and Physical Sciences University of Leeds West Yorkshire UK

**Keywords:** computer vision, medical image processing, object detection, surgery

## Abstract

Real‐time detection of surgical tools in laparoscopic data plays a vital role in understanding surgical procedures, evaluating the performance of trainees, facilitating learning, and ultimately supporting the autonomy of robotic systems. Existing detection methods for surgical data need to improve processing speed and high prediction accuracy. Most methods rely on anchors or region proposals, limiting their adaptability to variations in tool appearance and leading to sub‐optimal detection results. Moreover, using non‐anchor‐based detectors to alleviate this problem has been partially explored without remarkable results. An anchor‐free architecture based on a transformer that allows real‐time tool detection is introduced. The proposal is to utilize multi‐scale features within the feature extraction layer and at the transformer‐based detection architecture through positional encoding that can refine and capture context‐aware and structural information of different‐sized tools. Furthermore, a supervised contrastive loss is introduced to optimize representations of object embeddings, resulting in improved feed‐forward network performances for classifying localized bounding boxes. The strategy demonstrates superiority to state‐of‐the‐art (SOTA) methods. Compared to the most accurate existing SOTA (DSSS) method, the approach has an improvement of nearly 4% on mAP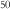 and a reduction in the inference time by 113%. It also showed a 7% higher mAP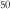 than the baseline model.

## Graphical abstract

Real‐time approach for accurate anchor‐free surgical tool detection by leveraging multi‐scale feature extraction and adding positional encoding in a transformer‐based detection architecture. A supervised contrastive loss optimizes representations of object embeddings, further. Improvement in both speed and accuracy compared to several SOTA methods is demonstrated.

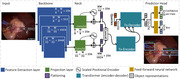

## 1 INTRODUCTION

Minimally invasive surgery (MIS) vision analysis has proved to be crucial in developing new technologies that can improve the outcome and performance of various minimally invasive procedures [1]. Vision analysis of surgical data could facilitate scene understanding by providing context and characteristics of the procedures [2, 3]. After the procedures, this information can be used in the feedback report for computer‐assisted diagnosis and automatic assessment of operative skills. During surgical procedures, vision analysis can be used in a real‐time decision support system for computer‐assisted detection and diagnosis. Additionally, with the latest MIS technology, human‐robot collaborative surgery can be achieved using vision analysis to automate specific tasks [4, 5, 6].

Current research has associated understanding of the surgical scene with descriptive solutions to domain‐related tasks. Some of the most relevant are depth estimation, phase recognition, tool recognition, detection, and tracking, and anatomy recognition and detection [7]. Although all of these tasks share some similar principles, the development of solutions for each of them requires different data types with different acquisition challenges [8]. Tool‐related tasks are the ones that have found the path less resistant to the data acquirement and hence, to prove concepts and develop complex solutions [9]. Therefore, they have stood out as pivotal for higher understanding acquisition and constrained the focus of this work to tool detection.

Object detection, in computer vision, is the component that extracts patterns from digital images or video frames and synthesizes the information in the classification and localization of specific objects [1, 3]. In surgical scenarios, challenges for the analysis are exacerbated by the nature of the surgical data [10]. Visual artefacts are commonly encountered since the surface of tools and tissues are reflective, there is a constant movement of tools and camera, the production of gases when cauterizing or cutting blurs the images, changes in the illumination produce shadows, there is occlusion of tools and tissues of interest, and fine details of the anatomies change from one patient to another. Scale variation and multi‐class classification are also important problems in a surgical scenario due to the high similarity among surgical tools and the constant forward and backward movement of the endoscopic camera. Finally, real‐time processing is critical since the system and surgeon's actions must be taken in real‐time, and any delay might compromise the patient's safety and incur surgical accidents.

Early surgical tool detection methods attempted to address some of these problems based on handcrafted filters. However, now their performance has been overcome by deep learning‐based detectors [8, 11]. Implementing these models shifted the research community's focus from hand‐crafted feature extractors to deep‐learning methods that allow the generation of optimal filters. These increase detectors' performance and complexity, bring new deep learning‐related challenges and expose others [2, 7]. For instance, receptive field constraints pose a trade‐off between the extraction of local and global features [8]. In the surgical scenario, both local and global features are needed to differentiate similar tools and tissues at different scales. Anchor dependency is another major issue in modern detectors [12, 13, 14]. The detectors with the best performance in medical and non‐medical data rely on pre‐defined anchor boxes. They represent a prior assumption about the size, aspect ratio, and location of objects in the image. It is particularly detrimental to the detector's performance in a surgical scenario with high variance in the objects' location, orientation and scale [10, 15]. To mitigate these problems, we considered that a multi‐scale analysis and an increased capability of contextualization are key components in developing an optimal solution. On top of this, the development of a tailored object representation space that solves ambiguities in the multi‐class classification task is yet to be presented. Thus, our contribution can be summarized as below:

- Generation of richer features through incorporating a Res2Net [16] as backbone, an architecture that makes local‐scale consideration for the extraction of features.
- Multi‐scale position encoding of two projected features maps extracted from the backbone to incorporate features at multiple scales in the self‐attention mechanism of the transformer. We call this new architecture our proposed “'dense transformer” (DTX) network and it is inspired by the DETR detector [14].
- Contrastive learning over the object representation of the surgical tools to encourage consistency and separability in the feature embeddings of the different classes.

## 2 RELATED WORK

For object detection (also called location detection), the development of new methods has been mainly driven by the research groups that have facilitated datasets with tool location annotations [10] since they provide the means for supervised training and validation of results. For instance, Sarikaya et al. [17] presents the ATLAS dataset for robotic MIS instrument detection in a mock environment. It provides an interesting and valuable framework for proving concepts in robotic MIS. However, its use in developing models for real‐world scenarios is limited. Jin et al. [18] presented the first Fast RCNN‐based model for instrument detection on real surgical scenarios by adding location annotation to 2532 frames of the m2cai16‐tool dataset. Although the reported performance of their model is low (5 FPS and 0.6 mAP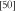), the m2cai16‐tool‐location dataset and deep learning techniques have significantly impacted the works forming state‐of‐the‐art (SOTA) in surgical tool detection. Zhang et al. [19] proposed a Fast RCNN‐based model and addressed the problem of anchor dependency with a modulated feature block to incorporate the anchor shape information in the generated feature maps from the backbone. A YOLO‐based model was presented by Choi et al. [20]. His work reported the fastest inference time of 48 FPS in the m2cai16‐tool‐location dataset but low performance for localization over preselected videos for validation. A similar single‐stage YOLACT++ [21] framework with multi‐scale fusion was used for an instance segmentation of tools in ROBUST‐MIS challenge dataset [22]. However, the developed method only enabled the presence of tools but not their class categories. Sai and Sinha [23] presented a multitask model for tool presence, detection (multi‐class), and phase classification based on a DSSD architecture (deconvolutional single shot detector). They explained how features from different parts of the architecture can be taken to solve different tasks and achieve improved performance regarding location inference. They did not report on the inference time, but based on the original DSSD paper [24], the speculated inference time is 15 FPS. Recently, Ali et al. [25] trained their model on the m2cai16‐tool‐location dataset under a semi‐supervised learning paradigm using a teacher‐student framework to address the data scarcity problem for multi‐class tool detection. Their results showed improved accuracy with 10% of the annotated data, but inference time was not reported. Zhao et al. [26] proposed a lightweight cascaded CNN architecture from coarse to fine. The first stage in a two‐stage detector was similar to a region proposal stage but with fixed‐sized regions. The second was a regression network of the surgical instrument tip region. They reported an inference time of nearly 24 FPS; however, they detected and tracked tip instruments without classification. Similarly, Liu et al. [15] proposed a method for tool location without classification over the ATLAS dataset and a relabelled version of the Endovis Challenge 2015 dataset. Also, they focused on anchor‐dependant methods using a compact stacked hourglass network that predicted the centre of the boundary box (but not multi‐class instruments) with high accuracy and speed (37 FPS).

Another MIS‐related dataset is the Cholec80 dataset [27], which includes phase and tool presence annotations for 80 videos of cholecystectomy. Vardazaryan et al. [28] proposed preserving spatial information with a fully convolutional neural network. It predicts instrument presence, and posteriorly, an analysis of the activation maps gives the instrument location. They used a subset of the Cholec80 dataset, selecting images with one instrument per frame since the analysis does not allow multi‐instance detection. In 2020, Shi et al. [29] at Shandong University took 4011 frames from the Cholec80 dataset and added spatial annotations on the tips of the tools for multi‐instance detection. They proposed a two‐stage detector, an attention‐guided convolutional neural network with coarse and refined modules, to achieve high inference time (55.5 FPS) and mAP (91.65%). Cholec80‐location subset was also used on a one‐stage detector by Yang et al. [30], adding modifications to the backbone and neck of the architecture. In the backbone, they used a GoshtNet architecture and cross‐stage partial connections to increase inference time and enhance the learning process. In the neck of the detector, they used a U‐Net and spatial pyramid pooling to address the multi‐scale problem. This work reported an mAP of 91.6% and a time inference of 38.5 FPS. However, there is no free access to this Cholec80‐location subset for a fair comparison in the tool detection and classification tasks, limiting the usability and reproducibility of the techniques explored in these works. In 2022, Kondo, S. [31] explored the use of a transformer for tool presence without location.

Although numerous studies have made notable advances in object detection for surgical instruments in MIS, existing approaches have only partially addressed the challenges of high accuracy and inference speed. Therefore, there is a need for a comprehensive solution that concurrently tackles these issues and enables the practical deployment of a real‐time tool detector in MIS settings with higher detection and localization performance. As detailed above most of the public datasets either have only presence (e.g. Cholec80) or lack labels for different surgical instrument types (e.g. Endovis Challenge 2015 dataset). Thus, in this work, we will evaluate our method on the m2cai16‐tool‐location dataset, which has been largely used for multi‐class tool detection and localization.

## 3 METHOD

**FIGURE 1:**
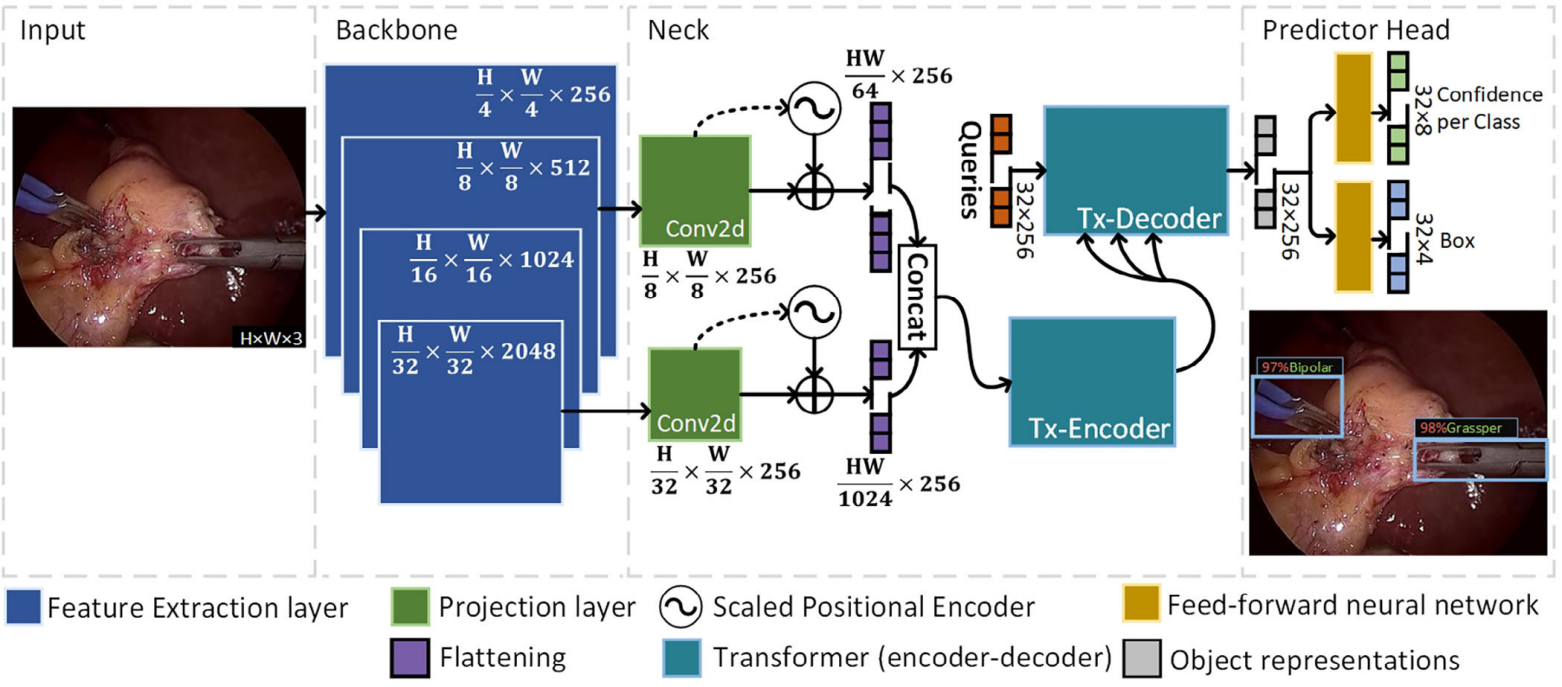
DTX network architecture. Our proposed DTX architecture uses a Res2Net [16] to extract feature maps at two different scales and forms a dense feature embedding by adding the projection layers (Conv2d) that set the same number of channels in each projection. Then our network, inspired by DETR [14], exploits the use of scaled positional encoders to locate the features from different projections under a common framework. Finally, the decoded object representations by the transformer go through two different feed‐forward neural networks for class and boundary box prediction.

In this work, we propose a new setup for the architecture and training of a multi‐scale transformer‐based detector (Figure 1) that incorporates Res2Net architecture as a backbone and extracts multi‐scale features maps (from two resolutions) addressing the limitation of small receptive fields and enhancing overall model robustness against scale changes of objects in the images. The extracted features from the backbone go through different 1×1 2D convolutional layers (Conv2d) that reduce the channel dimension to 256. They bring the feature maps from different resolutions to the same feature space. Thereby, global multi‐scale feature analysis is enabled in the transformer encoder (Tx‐encoder). Subsequently, the decoder of the transformer (Tx‐decoder) creates a set of object representations that are ultimately processed by two feed‐forward neural networks that predict the class and location of the objects. In addition to the Hungarian loss, we also proposed the integration of a contrastive loss (CL loss) in the training of the model. CL loss leverages the output of the TX‐decoder to encourage consistency and separability over the generated object representations. Below we provide a detailed description of the final network architecture and the combined loss functions used in this work.

### 3.1 Architecture details

**FIGURE 2:**
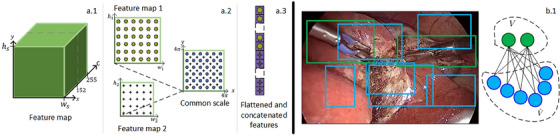
Feature map processing. (a) Embedded feature map structure after the projection layer. The position x and y of each feature are encoded in the first and second half of the channels, respectively. (b) The positions x,y of the feature maps are scaled by Equation (1) so the transformer can be aware of the location of the features under a common framework. (c) Input of the transformer: Flattened and concatenated features after positional encoder. Bipartite graph. (d) GT boxes in green form the set of vertices V, and predicted boxes in blue form the subset $\hat{V}$. Initially, all elements between sets are connected.

Similar to the recent DETR network [14], after the extraction of features from the backbone network, we use a transformer for learning reliable feature representations using self‐attention mechanisms. However, extra projection layers and concatenation of scales are added for the feature maps taken from the backbone. The projection layers (1×1 convolutional layers) reduce the channel dimension to 256, so there is a common feature space between scales (see Figure 2(a–c)). We then scale the positions $\left(x_{j},\;y_{j}\right)$ of the features at different scales such that the position of each feature is referenced to a common location (x,y) despite coming from different resolutions (see Figure 2(b). The position for each channel $c_{k}$, with k representing the index of the feature channel in the sine positional encoder, is calculated using Equation (1) where width and height are represented as $w_{s}$ and $h_{s}$, respectively, at scale s.

(1) $$\text{pos}\left(x_{j},y_{j},c_{k}\right)=\left\{\begin{array}{cc} \frac{4\pi }{w_{s}}x_{j} & k\in [0,152]\\ \frac{4\pi }{h_{s}}y_{j} & k\in [153,255] \end{array}\right.$$

Then the embeddings after positional encoding are flatted to a shape $\left(h_{s}w_{s},256\right)$, and these are concatenated along the first axes such as $(\sum_{s=1}^{s=2}\left(h_{s}w_{s}),256\right)$ is the final input shape to the transformer encoder (Tx‐encoder) (see Figure 2(c)). Within the Tx‐encoder, the multi‐head self‐attention modules focus the attention on the features from different scales that are more relevant to the final prediction. In this way, we leverage the transformer for performing attention to both local and global features. The transformer decoder (Tx‐decoder) takes a matrix of zeros as the query to initialize the decoding process. This query shapes the final output by assuming the maximum number of objects in the image and encrypting each object representation in 256 values. Finally, two feed‐forward neural networks make the final prediction. A one‐layer perceptron with a softmax activation function processes each object representation for its classification giving the highest probability to the detected object (or not‐object class). For the prediction of the boundary box of each object representation, a multi‐layer network (3 layers) infers the coordinates of the box (centre x, y, w, and h).

### 3.2 Loss functions

We introduce a contrastive loss function in addition to the loss function implemented in DETR [14]. We have a similar matching stage, but unlike DETR, we exploit the matching solution to incorporate the contrastive loss and jointly optimize it with the Hungarian loss.

#### 3.2.1 Matching stage

For each image, a $\hat{V}$ set is formed by the predictions of the model and a V set is formed by padding the objects in the ground truth (GT) such that both sets have the same number of elements. Each element $v_{i}$ in V contain $\left(c_{i},b_{i}\right)$ where $c_{i}$ is the class associated with the boundary box $b_{i}$ and the padded elements have a $c_{i}$ value of no‐object class (⌀). Similarly, the element ${\hat{v}}_{j}$ in $\hat{V}$ contain $\left({\hat{o}}_{j},{\hat{c}}_{j},{\hat{b}}_{j}\right)$ for the object representation, class and boundary box predicted by the model. All the elements in one set are connected to the elements in the second set to form the graph G, thus forming a bipartite graph (Figure 2(d)).

The comparison between the boundary boxes in the GT and the predictions are given by the box loss in Equation (2), where a weighted sum of the $L_{1}$‐norm and the generalized intersection over union (GIoU) are used. The matching costs (mc) of a connection (edge) in G is given by Equation (3), where ${\hat{b}}_{j}$ and $b_{i}$ are boundary boxes (predicted and GT), ${\hat{p}}_{j}\left(c_{i}\right)$ is the predicted probability of class i (the GT class) for the predicted box j, and $L_{box}$ the box loss function.

(2) $$L_{\text{box}}\left({\hat{b}}_{j},b_{i}\right)=\lambda_{1}\left(L_{1}\left({\hat{b}}_{j},b_{i}\right)\right)+\lambda_{2}\left(G\text{IoU}\left({\hat{b}}_{j},b_{i}\right)\right)$$

(3) $$mc_{ji}=L_{\text{box}}\left({\hat{b}}_{j},b_{i}\right)+\lambda_{3}\left(1-{\hat{p}}_{j}\left(c_{i}\right)\right)$$

The costs matrix CM is then calculated for all samples at indexes i and j by finding the matching cost between the elements of the prediction and the GT. Later, the Hungarian algorithm is used to find unique correspondences between the elements of the sets such that the sum of the matching costs of those correspondences is the minimum. It does that by finding the permutation of the rows in CM that minimize the trace of the matrix so, in the found permutation h, h(i) is the index j of the matched prediction to the element i in the GT.

#### 3.2.2 Hungarian loss

The Hungarian loss function [14] is then applied as shown in Equation (4), which is a weighted combination of the cross‐entropy loss and the defined box loss function.

(4) $$L_{H}\left(V,\hat{V}\right)=\sum_{i}-\lambda_{3}\ln\left({\hat{p}}_{h(i)}\left(c_{i}\right)\right)c_{i}+1_{\left\{c_{i}\neq ⌀\right\}}L_{\text{box}}\left({\hat{b}}_{h(i)},b_{i}\right)$$

ALGORITHM 1 **Supervised contrastive learning algorithm for multi‐class labels.**

**Table:** 

**Require**: Batch: B; Classes: C
$n_{c}=0$ ▹ number of classes
L=0
**for** c ∈C **do**
pos_samples = neg_samples = {}
**for** k∈{0 to len(B)} **do**
**for** i∈{0 to len(V[k])} **do**
**if** V[k].c[i] == c **then**
pos_samples $\leftarrow \hat{V}\left[k\right].o\left[h\left[k\right]\left(i\right)\right]$
**else**
neg_samples $\leftarrow \hat{V}\left[k\right].o\left[h\left[k\right]\left(i\right)\right]$
**end if**
**end for**
**end for**
P = N = {} ▹ positive & negative contrastive pairs
**for** o∈ pos_samples **do**
**for** $o^{\prime }\in$ neg_samples **do** $N\leftarrow (o,o^{\prime })$
**end for**
*pos*_*samples.pop (o)* ▹ Remove the reference from the list
**for** $o^{\prime }\in$ pos_samples **do** $P\leftarrow (o,o^{\prime })$
**end for**
**if** P==∅ **then** P←(o,o)
**end if**
**end for**
$n_{c}=n_{c}+1$
L=L+Eq5(P,N)
**end for**
$L_{\text{contrastive}}=L/n_{c}$

#### 3.2.3 Contrastive loss

We propose to add a complementary contrastive loss ($L_{\text{CL}}$) that is jointly optimized with the Hungarian loss in our final loss function. The use of $L_{\text{CL}}$ helps to cluster representations for each class while separating clusters of different classes. The proposed loss is a variation of the normalized temperature‐scaled cross‐entropy loss (NT‐Xent loss) presented in SimCLR [32]. The main difference is that the proposed CL loss can operate over a supervised paradigm leveraging the solution provided by the Hungarian algorithm. To do so, we look at the samples k in the batch B that contains ($V_{k}$, ${\hat{V}}_{k}$, $h_{k}$) for the GT, predictions, and optimal correspondences, and we aim to find all the positive P and negative N contrastive pairs for each class c in the batch as presented in algorithm 1. $P_{c}$ contains all the pairs of object representations $\left(o,o^{\prime }\right)$ such that their classes are equal, and $N_{c}$ contains all the pairs such that their classes are different. Note that $P_{c}$ avoids the self‐comparison, but when the number of representations related to a given class is equal to 1, the pair (o,o) added in $P_{c}$ to pull apart that representation from the rest of classes in the batch. Then Equation (5) shows the contrastive loss for each class using $P_{c}$ and $N_{c}$, it applies cosine similarity sim between the object representations.

(5) $$L_{{\text{CL}}_{c}}\left(P_{c},N_{c}\right)=-\log\frac{\sum_{\left(o,o^{\prime }\right)\in P_{c}}\text{exp}\left(\text{sim}\left(o,o^{\prime }\right)\right)}{\sum_{\left(o,o^{\prime }\right)\in \left(P_{c}\cup N_{c}\right)}\text{exp}\left(\text{sim}\left(oi,o^{\prime }\right)\right)}$$

The total contrastive loss is the average of all the contrastive losses per class in a given batch B with nc classes and size bs. Thus, the final loss L which is an equally weighted sum of the Hungarian loss and the contrastive loss, can be represented as:

(6) $$L\left(B\right)=\frac{\lambda_{4}}{bs}\sum_{k=0}^{bs}L_{H}\left(V_{k},{\hat{V}}_{k}\right)+\frac{\lambda_{4}}{nc}\sum_{c=0}^{nc}L_{{\text{CL}}_{c}}\left(P_{c},N_{c}\right).$$

## 4 EXPERIMENTS AND RESULTS

### 4.1 Dataset

We evaluate our architecture on the publicly available m2cai16‐tool‐location dataset [18] containing 2532 labelled frames from 15 videos of cholecystectomy procedures performed at the University Hospital of Strasbourg in France. To make our method comparable and reproducible, we have used the same split proposed in the original paper [18]. The final experimental dataset comprises 1405 images for training, 843 images for validation, and 563 images for testing (held‐out set). As Sahu [33] pointed out, this dataset poses an extra challenge to a solution for the multi‐class classification problem since it mirrors the imbalance appearing of the surgical tool during the operation. Therefore, the seven tool classes plus one extra for the background class were considered in the ground truth labels, and a discussion on how the implemented solution alleviates this problem is presented in the results section.

### 4.2 Experimental setup

#### 4.2.1 Data augmentation

All images were resized to 320×320 pixels. Six different geometric transformations were selected for data augmentation. During training, the transformations were randomly applied with a 33% probability each.

#### 4.2.2 Model configuration

The optimal hyper‐parameters for our model are reported in this section. However, a hyper‐parameters search grid is presented in the ablation study. The building blocks in the Res2Net50 architecture (the used backbone) were configured to split the feature maps into four sets of 26 channels each. In the neck of our architecture (see Figure 1), the feature maps that go through the projection layers were taken from layers 2 and 4 of the backbone. The number of queries that initialize the decoder process in the transformer was set to 32, and the number of layers in the encoder and decoder of the transformer to 6.

#### 4.2.3 Training setup

We build our model leveraging part of HuggingFace's Transformers repository [34] and making the pertinent changes to match the model's description presented in Section 3. During training, an AdamW optimizer with a step learning rate scheduler was added. The scheduler tracked and modified the learning rate from $1.0e^{-04}$ to $1.0e^{-06}$, with a factor of 0.5 at every 40 epochs. In addition, a stopping criteria tracking the validation loss was included in the experiment. It had a patience of 50 epochs and considered a minimum delta of $1.0e^{-0.5}$. We run all our code in a setting with multiple CPU processors provided by the Research Computing Team at the University of Leeds in their High‐Performance Computing facilities. The requested nodes provided 48GB system memory and an NVIDIA V100 32 GB graphic card.

#### 4.2.4 Evaluation metrics

We present and compare the performance of our model based on two widely used metrics called mean average precision (mAP) for object detection. For this metric, a threshold value is used to determine if detection is considered a true positive or a false positive based on the IoU (intersection over union) value ranging from [0.5:0.05:0.95] for overall mAP and at specific IoUs, e.g. [0.5] and [0.75]. The second metric reported is the inference time in frames per second (FPS).

### 4.3 Comparison with SOTA and baseline methods

In this section, we provide a comparison with state‐of‐the‐art methods used for detection tasks on the m2cai16‐too‐location dataset. Alongside this, we also present quantitative results on the baseline model and provide results for different architectural changes that have been proposed.

#### 4.3.1 Quantitative results

Tables 1 and 2 present the comparison of the SOTA methods for supervised surgical tool detection, anchor‐free methods in the literature and our propositions for overall mAP and AP for each class category, respectively. From Table 1, it is evident that our proposed approaches outperformed both the SOTA methods and other anchor‐free methods. For example our final model (DTX+MS+CL) has mAP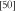 is 4% above the best SOTA method (DSSS), and nearly 7% higher than the baseline DETR. Our experiments also showed an additional improvement at mAP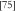 over the baseline with 0.572 compared to 0.524, which is 9% above. On the FPS, our method achieves 113% higher than the SOTA DSSS method and is only slightly lower than DETR‐baseline methods (4 FPS lower). Table 2 showed significant improvement in all class categories compared to the SOTA and the baseline DETR, regardless of the frequency with which each tool class appears in the dataset's images. Common (for example the grasper and hook) and rare (for example scissors and bipolar) tools are detected with high mAP, which suggests that the model focuses on relevant features from the images for the formation of the object representations associated with each class. For example compared to the most accurate method, DETR, our approach achieves 8%, 7.7%, 11%, 3%, 8.8%, 2.7%, and 5.2% respectively, for grasper, bipolar, hook, scissors, clipper, irrigator, and specimen bag.

**TABLE 1:** Quantitative results. Comparison of state‐of‐the‐art surgical tool detection methods, anchor‐free methods, and our proposed dense transformer (DTX) with and without multi‐scale and contrastive loss inclusions.

Model	mAP 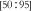	mAP 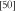	mAP 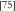	FPS	Backbone	Input size
**SOTA comparison**						
F. R‐CNN [18]	NA	0.631	NA	5	VGG‐16	NA
F. R‐CNN [19]	NA	0.696	NA	15*	ResNet101	NA
YOLO [20]	NA	0.722	NA	48	DarkNet19*	448×448
DSSS [23]	NA	0.912	NA	15*	ResNet101	320×320
F. R‐CNN+SSL [25]	0.468	0.902	0.462	15*	ResNet50‐FPN	NA
**Anchor free methods**						
FCOS [13]	—–	0.900	—–	12	ResNet50	450×450
DETR [14] (baseline)	0.520	0.886	0.524	**36**	ResNet50	320×320
**Our proposed approaches**						
DTX	0.536	0.926	0.557	35	ResNet50	320×320
DTX + MS	0.543	0.939	0.561	32	Res2Net50	320×320
DTX + MS + CL	**0.545**	**0.945**	**0.572**	32	Res2Net50	320×320

DTX, dense transformer (our method); contrastive loss, CL;MS, multi‐scale backbone

NA, not available; * This value was not officially reported by the author

**TABLE 2:** Quantitative results. Average precision (AP) comparison per class.

Method	Grasper	Bipolar	Hook	Scissors	Clipper	Irrigator	S. Bag
**SOTA comparison**							
Fast R‐CNN [18]	0.483	0.670	0.784	0.677	0.863	0.175	0.765
Fast R‐CNN [19]	0.541	0.695	0.868	0.739	0.842	0.416	0.771
YOLO [20]	0.893	0.324	0.932	0.666	0.903	0.424	0.914
**Anchor free methods**							
FCOS [13]	0.846	0.927	0.942	0.905	0.903	0.857	0.922
DETR [14] (baseline)	0.826	0.910	0.864	0.915	0.844	0.932	0.911
**Our proposed approaches**							
DTX (ours)	0.871	0.957	0.933	0.900	0.926	0.942	0.950
DTX + MS	0.891	0.955	0.955	**0.965**	**0.933**	0.921	0.956
DTX + MS + CL	**0.894**	**0.980**	**0.960**	0.945	0.919	**0.957**	**0.959**

DTX, dense transformer (our method); contrastive loss, CL; MS, multi‐scale backbone

#### 4.3.2 Qualitative results

**FIGURE 3:**
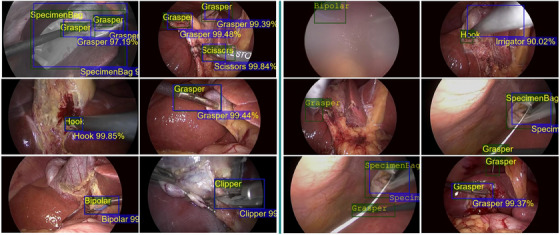
Qualitative results: Frames taken from the test set with their respective predictions. Predictions with the highest IoU are presented on the left, and predictions with the lowest IoU are presented on the right.

**FIGURE 4:**
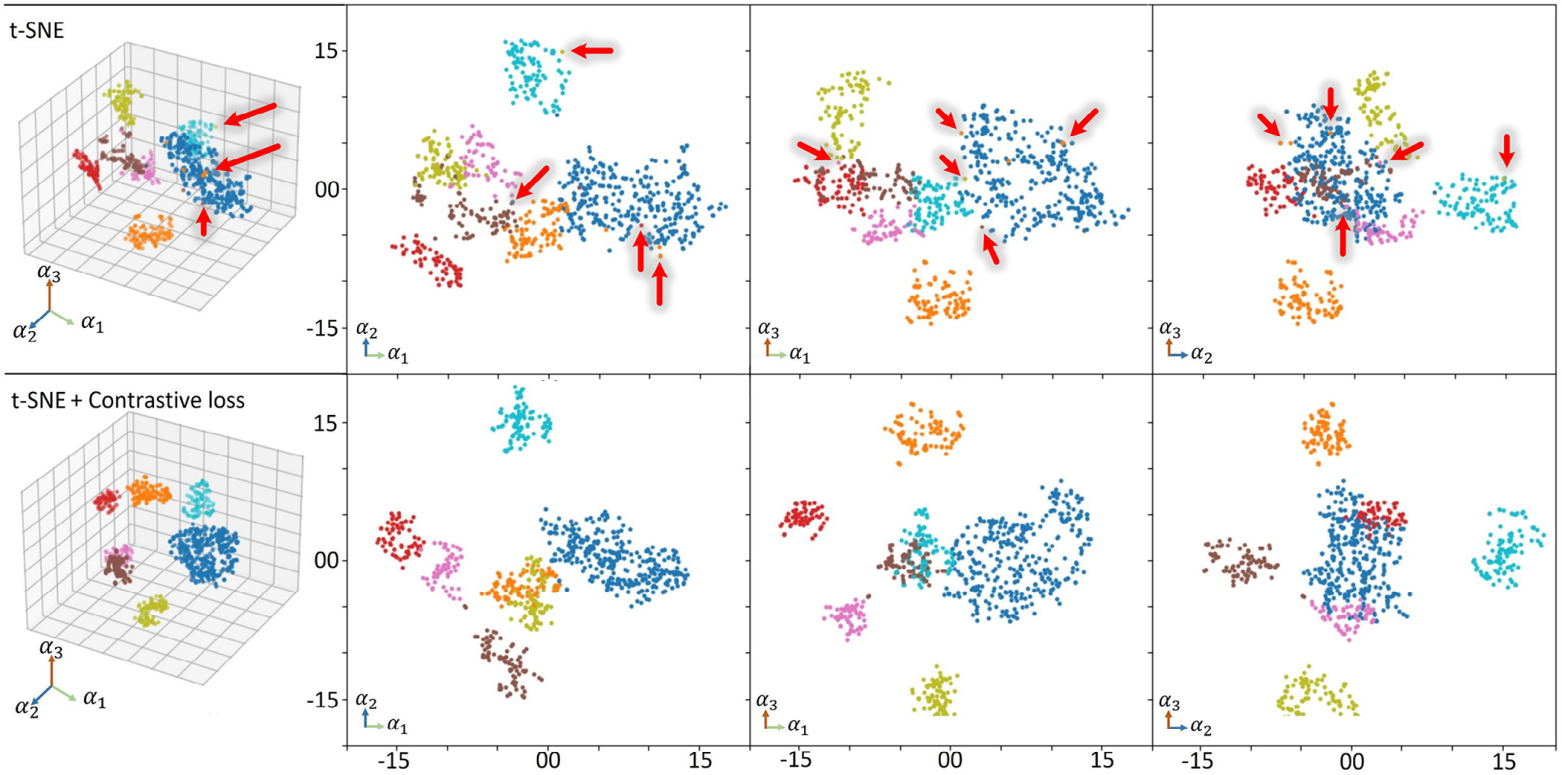
Object representation space. Dimensional reduction of the object representation space (TX‐decoder's outputs) using t‐SNE, each dot in the graphs represents a detected object by our model (DTX+MS). At the top, without contrastive learning (CL), the clusters for each class are barely separated, and some points are mislocated (see red arrows). This distribution is prone to hinter the performance of classifiers. However, at the bottom, we can clearly see how the integration of the CL alleviates this problem. There is a wide separation between clusters, and all the mislocated points were correctly rearranged.

**FIGURE A1:**
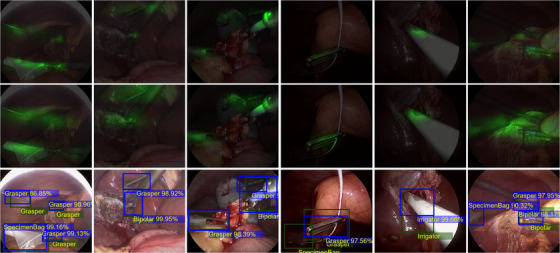
Attention maps for two scales. An attention map for the relevant object representations in each image is provided. This map was divided into the different scales that were used in our final network.

Figure 3 shows predictions from our proposed approach (DTX+MS+CL). The selected samples were the images with very low errors (on the left) and the images with the most significant errors (on the right). It can be observed that for the frames with optimal predictions, the predicted boxes (in blue) completely overlap the ground truth boxes (in green). However, for those with erroneous predictions (in the right), in most cases, either the object was not present (frame incorrectly labelled) or the object was incorrectly classified due to the fact that the intrinsic characteristics of the object are not present. In the second case, we can observe that our model makes a good guess by associating the object with a fairly similar tool. Figure 4 shows that the object representation space generated by our model in the decoder of the transformer is organized after implementing contrastive learning by maximizing the distance between the cluster of the classes and arranging misclassified objects. This adds up to the AP improvement presented in Tables 1 and 2, strongly suggesting that the error due to the misclassification of objects is considerably alleviated with our approach while boosting the performance. Having solved this problem, future efforts could be focused on developing methods that increase the precision of the predicted boundary box so that the value in the IoU is improved. Appendix Figure A1 shows the attention maps from the transformer's last layer in the decoder. Since we use feature maps at different scales, these images demonstrate how the relationship between the features in the regions of attention is present at different scales.

#### 4.3.3 Ablation study

The performance of models over the validation set for different network configurations (e.g. scales, feature layers, and feature maps) and combinations of relevant hyper‐ parameters (e.g. number of queries) is presented in Appendix Table A1. It can be observed that for different numbers of queries ranging from 32 to 100 queries, 32 queries boosted the performance of the model on the mAP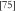 by 8.8% compared to using the number of queries proposed by DETR [14]. Our experiments also showed that a combination of four scales and 26 channels is the optimal backbone yielding 6.2% and 3.6% of improvement on the mAP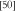 and mAP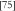, respectively. The number of layers in the encoder and decoder of the transformer shows that a network with six layers provided the best trade‐off between accuracy (0.866) and inference speed (FPS of 36). Finally, it can be observed that the inclusion of multi‐scale (MS) with the Res2Net backbone increases the mAP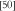 by 1.5% and boosts by 2% when CL is added, with only a slight decrease in FPS.

## 5 DISCUSSION AND CONCLUSIONS

Even though there are works in surgical tool detection in literature, these methods are widely built on anchor‐based methods, do not incorporate multi‐scale feature embedding for tackling variable tool sizes, and suffer from low speed [18, 19, 20, 23, 25]. Our approach using a transformer with the incorporation of multi‐scale feature selection is not only independent of anchors but also provides improved accuracy and inference time compared to SOTA methods in the literature. Utilizing the Res2Net backbone into our proposed dense transformer (DTX) enabled the inclusion of local and global features that can jointly tackle variations in the size of the objects and receptive field constraints. Our experiments showed improvement in almost all the tool categories by a large margin, up to 10.5%, compared to the baseline model (DETR [14]), which is the most consistent across the tool categories compared to any SOTA methods (Table 2). Further, we also showed that the incorporation of contrastive loss aids in minimizing inter‐class separation and maximizing intra‐class segregation, which helps to deal with closely similar‐looking tool categories (Figure 4 and Table 2). The less accurate predictions of our model are probably due to the fact that there are not enough intrinsic features of the object within those samples, and confusion might happen, for example misclassification of grasper and clipper (Figure 3). Consideration of features from previous frames could alleviate this problem and boost a more accurate prediction.

In conclusion, we proposed a transformer‐based surgical tool detection method introducing a novel multi‐scale feature assembly and incorporation of contrastive loss function utilizing information from the bipartite graph. The proposed model is anchor‐free and has near real‐time performance (32 FPS). To this extent, we also demonstrated the superiority of our approach compared to several SOTA approaches and other anchor‐free methods. The qualitative results also demonstrated the effectiveness of our model, with high‐quality predictions even in the challenging scenes. In our future work, we aim to leverage video temporal features to improve tool detection.

## AUTHOR CONTRIBUTIONS

**Gerardo Loza**: Conceptualization; methodology; investigation; data curation; software; validation; formal analysis; writing—original draft; writing—review and editing. **Pietro Valdastri**: Conceptualization; writing—review and editing; resources and supervision. **Sharib Ali**: Conceptualization; methodology; investigation; software; formal analysis; resources; writing—original draft; writing—review and editing; and supervision.

## CONFLICT OF INTEREST STATEMENT

The authors declare no conflicts of interest.

## Data Availability

Openly publicly available data has been used and referenced in the article. All data in the used repository do not contain any patient information and are fully anonymized.

## APPENDIX A

**TABLE A1:** Grid search for important hyper‐parameters and ablation study.

Model	mAP	mAP	FPS	Queries
DETR	0.901	0.552	36	32
DETR	0.896	0.507	36	64
DETR	0.857	0.483	36	100

Model	mAP	mAP	FPS	Layers Tx‐e,Tx‐d
DETR	0.895	0.550	36	[6,6]
DETR	0.837	0.470	38	[5,5]
DETR	0.856	0.450	41	[4,4]
DETR	0.774	0.401	45	[3,3]
DETR	0.715	0.220	49	[2,2]
DETR	0.624	0.153	51	[1,1]

Model	mAP	mAP	FPS	scales,feats
DETR + MS	0.908	0.488	32	[4,26]
DETR + MS	0.905	0.488	26	[6,26]
DETR + MS	0.924	0.521	24	[8,26]
DETR + MS	0.899	0.443	21	[8,14]

Model	mAP	mAP	FPS	feature maps
DTX (ours)	0.927	0.581	36	[3,4]
DTX	0.930	0.590	36	[2,4]

Model*	mAP	mAP	FPS	
DTX (ours)	0.926	0.557	**36**	
DTX + MS	0.939	0.561	32	
DTX + MS + CL	**0.945**	**0.572**	32	

* These experiments use the best hyper‐params from grid search

DTX, dense transformer (our method); contrastive loss, CL

MS, multi‐scale backbone
